# The Association of Dental Faculties

**Published:** 1889-09

**Authors:** 


### The Association of Dental Faculties.

The annual meeting of the Association of Dental College Faculties was held at Saratoga, August 5, and the two subsequent days. A much larger number of colleges was represented than ever before, and a deeper interest in the work of the association was manifested than at any former meeting.

Quite a number of important educational questions were brought forward for consideration, and they were discussed very fully; but while there was considerable diversity of opinion on many points, and the discussion conducted with earnestness and vigor, yet it was done by each one with a very commendable regard and respect for the opinions and views of others. A spirit of conciliation was manifestly entertained by all present.

More was done for the real elevation and progress of dental education than at any former meeting. Perhaps the action had, of the greatest significance, was that in reference to the lengthening of the required terms by the colleges for graduation.

A resolution was passed by more than a two-third vote that all colleges having membership in this association shall at and after the Session of 1891–92 require three terms of not less than five months each for graduation. This will undoubtedly be recognized as the longest stride that has ever been taken at one effort in behalf of dental education. Nowhere have we heard any thing but approval of this action. As an indication in this direction we are glad to note that both the American Dental Association, and the Association of Boards of Dental Examiners of the different States, both being in session at the same time; by a unanimous vote sanctioned the action of the Association of Dental Faculties, in respect to the resolution just referred to. Other proceeding was had of which we would be glad to make note here, but perhaps it is not well to further anticipate the proceedings of this body, which we hope to give in full in the next mumber of the REGISTER.

